# Transcorneal electrical stimulation restores DNA methylation changes in retinal degeneration

**DOI:** 10.3389/fnmol.2024.1484964

**Published:** 2024-12-05

**Authors:** Ben Yi Tew, Gerald C. Gooden, Pei-An Lo, Dimitrios Pollalis, Brandon Ebright, Alex J. Kalfa, Alejandra Gonzalez-Calle, Biju Thomas, David N. Buckley, Thomas Simon, Zeyi Yang, Ege Iseri, Cody L. Dunton, Vadim Backman, Stan Louie, Gianluca Lazzi, Mark S. Humayun, Bodour Salhia

**Affiliations:** ^1^Department of Translational Genomics, Keck School of Medicine, University of Southern California, Los Angeles, CA, United States; ^2^Department of Ophthalmology, Keck School of Medicine, USC Roski Eye Institute, University of Southern California, Los Angeles, CA, United States; ^3^USC Ginsburg Institute for Biomedical Therapeutics, University of Southern California, Los Angeles, CA, United States; ^4^Department of Clinical Pharmacy, Alfred E. Mann School of Pharmacy and Pharmaceutical Sciences, University of Southern California, Los Angeles, CA, United States; ^5^Department of Biomedical Engineering, University of Southern California, Los Angeles, CA, United States; ^6^Institute for Technology and Medical Systems, University of Southern California, Los Angeles, CA, United States; ^7^Department of Biomedical Engineering, Northwestern University, Evanston, IL, United States; ^8^Department of Electrical and Computer Engineering, University of Southern California, Los Angeles, CA, United States

**Keywords:** retina, retinal degeneration, electrical stimulation, DNA methylation, epigenomics

## Abstract

**Background:**

Retinal degeneration is a major cause of irreversible blindness. Stimulation with controlled low-level electrical fields, such as transcorneal electrical stimulation (TES), has recently been postulated as a therapeutic strategy. With promising results, there is a need for detailed molecular characterization of the therapeutic effects of TES.

**Methods:**

Controlled, non-invasive TES was delivered using a custom contact lens electrode to the retinas of Royal College of Surgeons (RCS) rats, a model of retinal degeneration. DNA methylation in the retina, brain and cell-free DNA in plasma was assessed by reduced representation bisulfite sequencing (RRBS) and gene expression by RNA sequencing.

**Results:**

TES induced DNA methylation and gene expression changes implicated in neuroprotection in the retina of RCS rats. We devised an epigenomic-based retinal health score, derived from DNA methylation changes observed with disease progression in RCS rats, and showed that TES improved the epigenomic health of the retina. TES also induced DNA methylation changes in the superior colliculus: the brain which is involved in integrating visual signaling. Lastly, we demonstrated that TES-induced retinal DNA methylation changes were detectable in cell-free DNA derived from plasma.

**Conclusion:**

TES induced DNA methylation changes with therapeutic effects, which can be measured in circulation. Based on these changes, we were able to devise a liquid biopsy biomarker for retinal health. These findings shed light on the therapeutic potential and molecular underpinnings of TES, and provide a foundation for the further development of TES to improve the retinal health of patients with degenerative eye diseases.

## Introduction

Retinal degeneration is a major cause of irreversible blindness. It is projected that the incidence of retinal degenerative diseases will double by 2050 (Rein et al., 2009). These diseases can affect any part of the retina but are typically characterized by the loss of highly differentiated cells within the neurosensory retina, such as photoreceptors, as well as the retinal pigment epithelium (RPE) that underlies the retina (Fleckenstein et al., 2021). The most common retinal degenerative diseases are age-related macular degeneration (AMD) and retinitis pigmentosa (RP). Genetic mutations, family history, and environmental or pathologic damage to the retina are all factors that can lead to progressive retinal disease. Currently, multiple treatment modalities are used with varying levels of success to manage patients with different degrees of vision loss (Fleckenstein et al., 2021). Although pharmacological therapies aim to slow disease progression, there is still no cure for many patients suffering from retinal degeneration. Historically, electrical and magnetic stimulation of the retina has been limited to rehabilitative devices like the Argus II retinal prosthesis, which uses electrical stimulation to bypass damaged photoreceptors and activate remaining retinal neurons for partial vision restoration in patients with near-total blindness, but requires invasive surgery to be implanted (Humayun et al., 2012).

Stimulation with controlled low-level electrical fields delivered non-invasively has recently been postulated as a therapeutic strategy to protect the retina and other ocular tissue (Agadagba et al., 2023). Specifically, transcorneal electrical stimulation (TES) facilitated by placing a stimulating electrode on the surface of the eye or the eyelid has been shown to have a variety of effects on the retina, ranging from promoting survival of axotomized retinal ganglion cells to rescue of photoreceptors in animal models (Morimoto et al., 2007; Schatz et al., 2012; Henrich-Noack et al., 2013). Despite some promising observations, the effect of these electrical fields has been inconsistent in articles published to date (Jassim et al., 2021; Rahmani et al., 2013). In human clinical trials, TES was shown to have modest transient effects or no effects on the retina in trials of RP (Jolly et al., 2020; Schatz et al., 2017), AMD (Shinoda et al., 2008), and retinal artery occlusion (RAO) (Naycheva et al., 2013). Moreover, there is insufficient molecular characterization as to how TES exerts its therapeutic effects. While molecular changes at the level of the transcriptome have been reported (Tao et al., 2016; Willmann et al., 2011; Yu et al., 2020), a comprehensive analysis of epigenomic changes and how they relate to transcriptional changes in response to TES has not yet been reported.

The goal of this work was to elucidate the electrical parameters that can cause epigenetic changes in the retina to prevent or delay retinal degeneration in a retinal dystrophic animal model using Royal College of Surgeons (RCS) rats (Ryals et al., 2017). We have recently shown that controlled, non-invasive TES promotes the survival of photoreceptors in RCS rats, where TES at 100 μA, but not 20–50 μA, decreased retina hypopigmentation by funus autofluorescence and increased photoreceptor counts (Gonzalez Calle et al., 2023). In the current study, we show that TES induced treatment-dependent and sex-specific DNA methylation changes in the retina that were associated with potentially protective neuronal pathways. To determine if TES-induced DNA methylation changes were associated with improved retinal health, compared to sham-treated controls, we developed an “epigenomic score” by quantifying the DNA methylation changes in a well-defined system of natural disease progression in RCS rats. This epigenomic score increased with disease progression but decreased after TES, and was found to be indicative of improved retinal health. Epigenomic score-based retinal analyses revealed sex-specific differences in TES responses. Finally, we also showed that TES-induced retinal DNA methylation changes were detectable in circulating plasma cell-free DNA (cfDNA), highlighting the potential for monitoring treatment response using liquid biopsies.

## Materials and methods

### Animal experiments

RCS rats, which are an established model for retinal degeneration due to a *Mertk* gene mutation, were used in this study (Strauss et al., 1998). All animals were maintained on a daily 12-h light/day cycle before experiments. The care and use of the rats was conducted in accordance with the regulations and guidelines of the Institutional Animal Care and Use Committee (IACUC) of the University of Southern California (USC) (protocol #21014) which is consistent with the National Institutes of Health (NIH) Guidelines for the Care and Use of Laboratory Animals, and the Association for Research in Vision and Ophthalmology (ARVO) Statement for the Use of Animals in Ophthalmic and Vision Research.

### *In vivo* transcorneal electrical stimulation

To assess the effects of TES, the right eye (OD) of each RCS rat was subjected to stimulation for 2 h once a week, starting from postnatal day 21 (P21) as previously described (Gonzalez Calle et al., 2023). Animals underwent TES treatment at P21, P28, P35, P42, P49, and P56. Electrode configuration and TES setup was designed based on computational modeling and verified by electrophysiological recordings of the superior colliculus (Gonzalez Calle et al., 2023; Iseri et al., 2024). A 3 mm platinum stimulating ring electrode was placed on the right cornea, with a subcutaneous ground electrode positioned temporally. The eyes were stimulated with a symmetric charge-balanced cathodic-first biphasic stimulation with a pulse duration of 10 ms and stimulus frequency of 6 Hz. Stimulation was given at low or high current amplitudes or with no current as a sham control in the following treatment groups: Sham (n = 7 males/5 females), Low (20–50 μΑ) (n = 14 males/6 females), and High (100–150 μΑ) (n = 16 males, 4 females) (Supplementary Table S1) (Gonzalez Calle et al., 2023; Iseri et al., 2024). The range of 20–150 μA was chosen because 20 μA was previously shown to be the minimum current required to elicit a response by superior colliculus electrophysiology, while currents exceeding 150 μA had a risk of damaging the eye based on the Shannon safety curve (Gonzalez Calle et al., 2023). Biphasic, cathodic-first pulses with a pulse width of 10 ms were used. No electrode was placed on the contralateral left eye, which was used as an unstimulated control. Prior to each stimulation session, the rats were anesthetized with ketamine (80 mg/kg) and xylazine (8 mg/kg) by intraperitoneal injection, and their body temperature was maintained at 37°C using a water heating pad. Continuous monitoring of heart rate and respiration was carried out throughout the procedure. The rats were euthanized at P60 by either Euthasol (pentobarbital + phenytoin), or isoflurane overdose, and death was confirmed with a secondary measure (heart snip). Ocular and brain tissues were collected for further analysis. The retina of the unstimulated eye, the retina of the stimulated eye, and the superior colliculus were dissected and flash-frozen. Both the left and right superior colliculus regions were dissected. Whole blood was collected at P60 by cardiac puncture during euthanasia. Blood was collected in the presence of EDTA to prevent coagulation. To study the effects of natural retinal degeneration in RCS rats, untreated RCS rats were euthanized at 5 different time points, P21, P35, P42, P49, and P60 (n = 3/4 per group, per sex) (Supplementary Table S2). The same set of tissues was collected from these rats after euthanasia.

### DNA and RNA extraction

Rat retina and brain tissues were flash-frozen in liquid nitrogen directly after dissection. DNA and RNA were simultaneously extracted using the AllPrep DNA/RNA Mini Kit (Qiagen), with tissue homogenization performed using the Bullet Blender (Next Advance). DNA and RNA were quantified using TapeStation genomic DNA or RNA ScreenTape on the 4,200 TapeStation (Agilent).

Blood was separated by centrifugation at 1,900×*g* and the plasma layer was isolated. The plasma was spun again at 5,000×*g* to remove any residual debris. Plasma cfDNA was isolated using the MagMAX Cell-Free DNA Isolation Kit (ThermoFisher) according to the manufacturer’s instructions. cfDNA was quantified using TapeStation D1000 High Sensitivity DNA ScreenTape (Agilent).

### Bisulfite sequencing

Reduced representation bisulfite sequencing (RRBS) was performed using the Ovation RRBS Methyl-Seq kit (Tecan) according to the manufacturer’s instructions. Briefly, genomic DNA was digested with MspI, subjected to adapter ligation, and treated to undergo final repair. Libraries were bisulfite converted and PCR amplified.

Whole genome bisulfite sequencing (WGBS) was performed on plasma cfDNA as previously described (Buckley et al., 2022). Due to limited plasma volume, multiple samples had to be pooled to achieve the minimum amount of DNA required for sequencing (n = 3–4 per pool) (Supplementary Table S3). Briefly, library preparation was performed with the Ovation Ultralow Methyl-Seq Library System (NuGen) using the manufacturer’s suggested protocol. Bisulfite conversion was performed on the libraries with EpiTect Fast DNA Bisulfite Kit (Qiagen), and the libraries were PCR amplified.

All libraries were quality-checked on the TapeStation D1000 High Sensitivity DNA ScreenTape. Paired-end sequencing was performed on final library products using the NovaSeq 6,000 platform (Illumina). After demultiplexing and adapter trimming, alignment to rn6 and methylation calling was performed using Bismark (Krueger and Andrews, 2011). Differentially methylated regions (DMRs) were identified with metilene (Juhling et al., 2016). The resulting DMR lists were filtered to contain only statistically significant DMRs (*p*-value <0.05) as determined using the Mann–Whitney-U test by metilene. Genes associated with DMRs were identified and analyzed using the Core Analysis workflow of Ingenuity Pathway Analysis (IPA) (Qiagen). Heatmaps were generated with the ComplexHeatmap package in R (Gu et al., 2016). Hierarchical clustering was performed using Manhattan distance and Ward’s method.

The epigenomic score, devised to quantify retinal health after TES, was derived from an epigenomic signature that correlated with the progression of retinal degeneration from untreated P21-P60 RCS rats. Min-max normalization was performed on the average beta values across the regions of interest, with the min/max values set to those from P21/P60 for hypermethylated regions. This was reversed for hypomethylated regions. TES-treated samples were normalized using the same parameters. Student’s *t*-tests were used to determine statistical significance between comparisons.

### RNA sequencing

RNA sequencing libraries were prepared using the NEBNext Ultra II Kit together with the rRNA Depletion Kit v2 and Unique Dual Index UMI Adapters (NEB). Equimolar pools of libraries were created and paired-end sequencing was performed on the Illumina Novaseq 6,000 platform for an average read length of 150 bp.

Sequencing data was aligned using STAR and read counting was performed by htseq-count. Differential gene expression was performed using the DESeq2 package in R (Love et al., 2014). Filtered differential expression gene lists were uploaded to IPA (Qiagen) and analyzed using the Core Analysis workflow.

## Results

### TES induces DNA methylation and gene expression changes in retina associated with neuronal pathways

**Figure 1:**
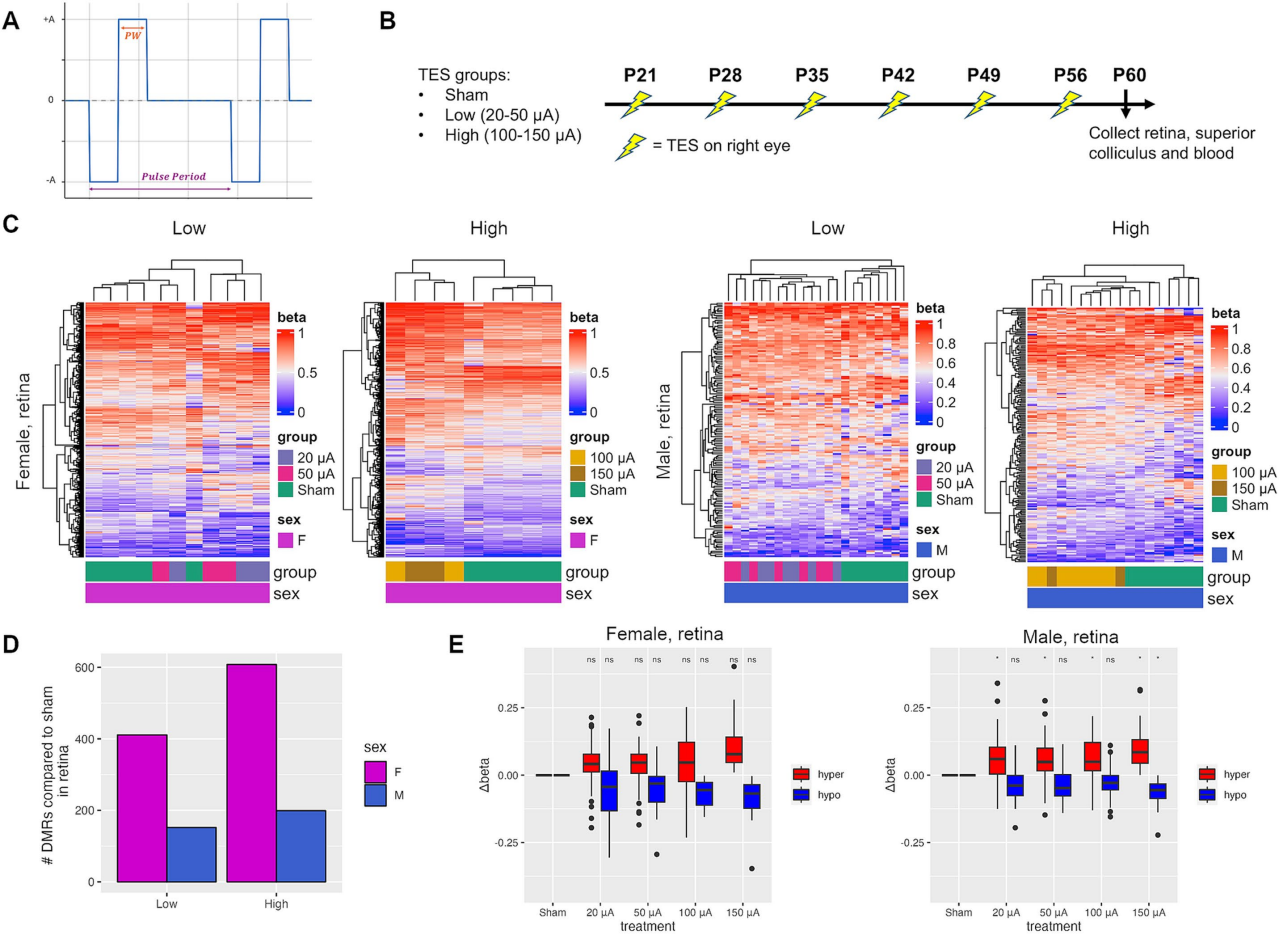
TES induces treatment-dependent and sex-specific DNA methylation changes in the retina. (A) A current-controlled rectangular biphasic waveform was used for continuous stimulation of rat retinas. A pulse period of 1/6 s (6 Hz), a phase width (PW) of 10 milliseconds and a range of amplitudes (±A) for low and high stimulation were used. The cathodic and anodic are symmetric and charge-balanced. (B) Experimental setup of TES treatment in RCS rats. (C) Heatmaps of differentially methylated regions (DMRs) identified between sham and TES-treated rats at low (20–50 μA) and high (100–150 μA) current amplitudes for females and males separately. (D) Number of DMRs identified between sham rats and those in each treatment group. (E) Change in methylation value (Δbeta) for selected hyper- and hypomethylated DMRs that displayed a treatment-dependent effect. Asterisks denote a *p*-value <0.05 when compared to sham controls using Student’s t-tests.

It is well established that RCS rats develop spontaneous retinal degeneration beginning at P21 and become blind by P60 (Ryals et al., 2017; Gonzalez Calle et al., 2023). We have previously designed a setup that can deliver TES to the retina, utilizing biphasic stimulus pulses of long pulse duration, which was shown to improve photoreceptor survival in the retina at 100 μA (Gonzalez Calle et al., 2023; Iseri et al., 2024). Here, a current-controlled rectangular biphasic waveform was used for continuous stimulation, with a pulse period of 1/6 s (6 Hz), a phase width (PW) of 10 milliseconds and amplitudes ranging from 20–50 μA for low and 100–150 μA for high stimulation (Figure 1A). RCS rats were treated with TES weekly from P21 to P56, for a total of 6 stimulations (Figure 1B) (Gonzalez Calle et al., 2023). TES was given at low (20–50 μA) and high (100–150 μA) current amplitudes, where low amplitudes were previously shown to promote a modest but non-significant improvement of the retina, while retinas stimulated with high currents had significant improvements as assessed by fundus autofluorescence and photoreceptor survival (Gonzalez Calle et al., 2023). Hierarchical clustering demonstrated the separation of differentially methylated regions (DMRs) identified between sham controls and treated retinas in both male and female rats at P60 (Figure 1C). There was an average of 3 times more DMRs in females compared to males (Figure 1D). The number of DMRs increased more steeply in females compared to males with treatment intensity, increasing by 48% to 608 DMRs between low and high current groups for females compared to a 31% increase in males to 199 DMRs (Figure 1B). Overall, there were more hypermethylated (57.2% in females; 61.2% in males) than hypomethylated DMRs (Supplementary Figure S1A). A subset of the DMRs had differential methylation that correlated with TES amplitude in both male and female rats (Figure 1E). More DMRs were also found in open sea regions in both male and female rats (Supplementary Figure S1). An average of 15.9 and 8.3% of DMRs occurred in CpG islands for females and males respectively, while 70.1 and 77.6% occurred in open sea regions. There was a small degree of overlap of DMRs between low and high current amplitudes, ranging from 7.0 to 9.3% (Supplementary Figure S2A). There was an average of 17.1% overlap between retinas from treated males and females (Supplementary Figure S2B), consistent with a sex-specific response due to TES.

**Figure 2:**
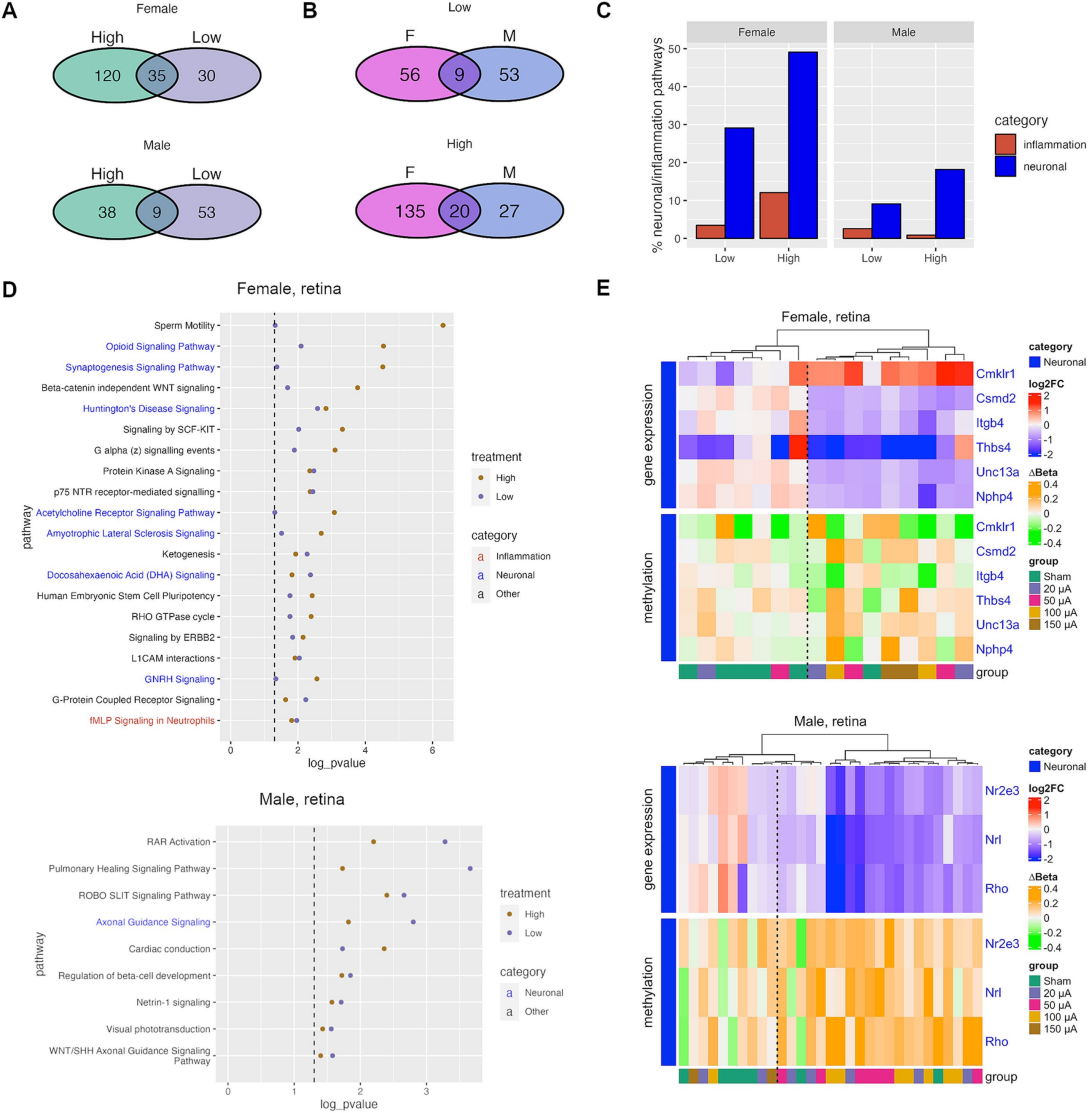
TES-induced DNA methylation changes are associated with neuronal and inflammation pathways. (A) Venn diagram showing the shared and unique canonical pathways identified through IPA analyses for the treatment groups. (B) Venn diagram showing sex-specific and unique IPA pathways for each treatment group. (C) Percent of all IPA pathways that fall under the neuronal or inflammation-related IPA pathway categories that were identified after TES treatment. (D) The top IPA pathways that were significantly altered for both low and high treatment groups. Dotted lines indicate a *p*-value <0.05, or −log10(*p*-value) > 1.3. (E) Heatmaps of log2 fold change (gene expression) and ∆beta (methylation) relative to the sham group of top genes that were treatment-dependent.

To determine the potential functionality of DMRs induced by TES, Ingenuity Pathway Analysis (IPA) was performed on genes associated with the DMRs. Of the pathways identified in the low current groups, 53.8% (35/65) compared to 22.5% (9/62) were also identified in the high current groups for females (Figure 2A). However, many fewer pathways were common between low (14.5%, 9/62) and high (19.1%, 9/47) current groups in males (Figure 2A). These data support the notion that TES induces many more amplitude-dependent changes in females compared to males. When it comes to overlapping pathways between males and females, in low current groups only 14.5% (9/62) of pathways in males and 13.8% (9/65) of pathways in females were common (Figure 2B). In high current groups, 42.5% (20/47) of pathways in males compared to 12.9% (20/155) of pathways in females were found in common (Figure 2B). These data demonstrate that the molecular changes in response to TES vary more dramatically between male and females at lower amplitudes compared to higher amplitudes. The data support the conclusion that TES induces more amplitude-dependent changes in females, with a higher overlap between low and high current pathways. In contrast, the molecular changes in males are more distinct across amplitudes, especially at lower currents. Additionally, cross-gender comparisons reveal that at low amplitudes, pathway overlap is minimal, reinforcing the idea of sex-specific responses. However, at higher amplitudes, the convergence of molecular pathways suggests a more synchronized response between males and females. This indicates that the impact of TES is both sex- and amplitude-dependent, with greater sex-specific changes at lower currents.

To better understand the pathways affected by TES, we analyzed significant pathway categories and found many related to neuronal function and inflammation across sexes and current groups (Figure 2C). Of the 55 neuronal pathways in IPA, 16 (29.1%) and 27 (49.1%) were found in the low and high current groups, respectively, in females. In males, 5 (9.1%) and 10 (18.2%) of neuronal pathways were found for low and high current groups, respectively. This suggests that TES modulates neuronal pathways in the retina, with a greater number of pathways impacted at higher amplitudes, particularly in females, indicating a stronger amplitude-dependent effect in females compared to males. Of the 116 inflammation related pathways in IPA, 4 (3.4%) and 14 (12.1%) were found in low and high currents, respectively, in females, and 3 (2.6%) and 1 (0.9%) were found in low and high currents, respectively, in males. These findings suggest that TES modulates inflammation-related pathways in an amplitude-dependent manner in females, with higher currents impacting a greater number of pathways. In contrast, the limited number of inflammation-related pathways in males indicates that TES may have a less pronounced effect on these pathways, regardless of the current amplitude. Key pathways, both common (Figure 2D) and unique (Supplementary Figure S3) to each treatment group are shown in Figure 2D were identified for females and males separately.

To assess the functional impact of the epigenetic changes induced by TES and explore whether neuronal pathway alterations might have protective effects, RNA-seq was performed on matching retinas. Differentially expressed genes, that also exhibited differential methylation, were identified between sham controls and each treatment group. Of the genes identified, 6/24 and 3/5 were associated with neuronal signaling in females and males, respectively (Figure 2E). This included genes associated with pathways identified previously (Figure 2D), such as *Thbs4* and *Unc13a* in the synaptogenesis pathway and *Rho* in the visual phototransduction pathway. TES downregulated genes known to cause neuronal diseases (*Nphp4*, *Unc13a*) (Placek et al., 2019), and also downregulation pro-apoptotic genes (*Thbs4*) (Rahman et al., 2020), supporting the assertion that TES induces neuroprotective changes by downregulating genes that are potentially neurodegenerative. Additionally, there was decreased expression of genes associated with retinal development (*Csmd2*, *Nrl*) (Cheng et al., 2004; Gutierrez et al., 2019), including some associated with rod differentiation (*Rho*, *Nr2e3*) (Oh et al., 2008). These data demonstrate the potential ability of TES to induce DNA methylation and gene expression changes functionally linked to promoting neuronal protection in both males and females. Interestingly, there were too few differentially expressed genes with methylation changes in inflammation-related pathways, suggesting that the epigenetic regulation of these pathways may be limited or occur independently of gene expression changes. Additionally, unsupervised clustering shown in Figure 2E using both expression and methylation data yielded two primary clusters consisting of either predominantly sham (left cluster) or TES-treated retinas (right cluster). However, several TES-treated retinas grouped with the sham cluster, suggesting that some retinas—particularly those in the low-current group—exhibited minimal expression and methylation changes in response to TES. This could be attributed to variability in treatment delivery. These findings align with our previous study, which demonstrated that only high-current stimulation (100 μA) induced significant phenotypic changes in the retina (Gonzalez Calle et al., 2023).

In addition to comparing the retinas of sham controls to TES-stimulated rats, methylation values from the unstimulated contralateral left eye were compared to the stimulated right eye of each individual rat. A similar treatment intensity-dependent effect of TES was observed, with the largest number of DMRs identified in the high current group (Supplementary Figure S4A). Sex-specific differences were also observed, with females exhibiting more methylation changes as compared to males. Interestingly, fewer DMRs were identified when comparing the stimulated eye to the contralateral eye, compared to sham vs. TES, suggesting that TES could have an indirect effect on the contralateral eye. IPA analysis of the DMR-associated genes of the TES-treated right eye versus contralateral left eye was performed (Supplementary Figure S4B). The top 20 canonical pathways were consistent between the sham vs. TES and TES-treated right vs. contralateral left eye comparisons for each individual rat, highlighting the consistent nature of TES-induced methylation.

### Epigenomic changes during retinal degeneration in RCS rats similar to changes observed after TES

**Figure 3:**
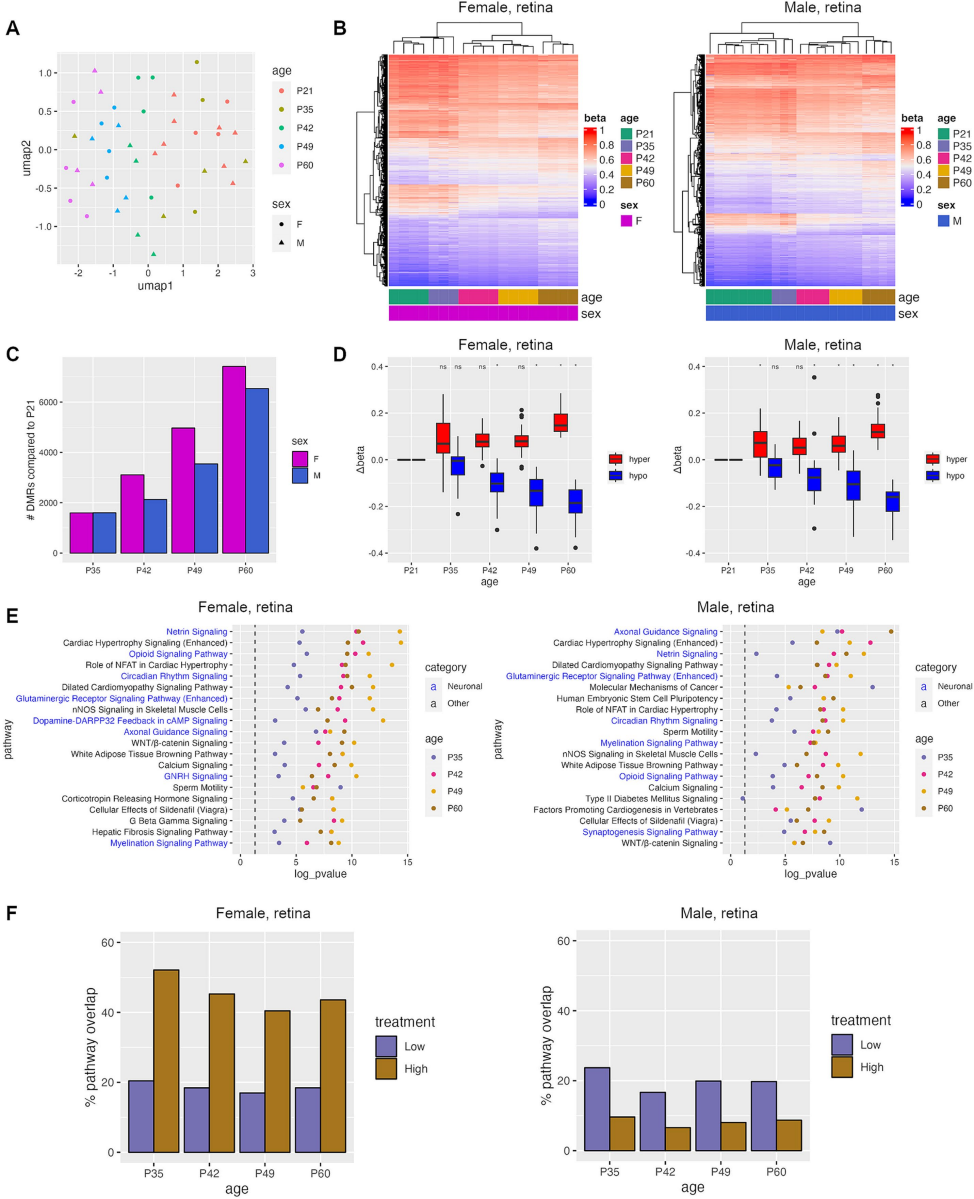
Progressive DNA methylation changes occur during natural retinal degeneration in RCS rats. (A) Umap plot showing the clustering of samples based on the top 100 k most variable CpGs. (B) Heatmaps of top DMRs identified between P21 and P60. (C) Number of DMRs identified between P21 and each other age group. (D) Change in Δbeta for hyper- and hypomethylated DMRs identified in retinas between P21 and samples from the specified time points. Asterisks denote a *p*-value <0.05 when compared to sham controls using Student’s *t*-tests. (E) Top IPA pathways that were common between all four age groups. (F) Percent of pathways that were identified during natural disease progression that were also identified after TES treatment.

In order to determine whether TES has a therapeutic effect on the retina, we first analyzed whether TES-induced DNA methylation changes resembled a healthier retina at a younger age in rats. To do this, we examined DNA methylation changes that occur with disease progression in RCS rats by analyzing RRBS data on retinas at 5 different ages (P21, P35, P42, P49, and P60). UMAP clustering showed similar methylation profiles between P21 and P35 retinas, and separation with increasing age beyond P42 regardless of sex (Figures 3A,B).

Differential methylation analysis was performed by comparing P21 (pre-degenerated) retinas to all other age groups, revealing an increase in the number and the delta beta of DMRs as the retina degenerates with age (Figures 3C,D). Most of the DMRs identified when retinal degeneration was present at P42 and older were retained through P60 (Supplementary Figure S5A). Overall, there were more unique DMRs than overlapping DMRs when comparing male and female rat retinas at different ages, but the frequency of overlapping DMRs between male and female rat retinas increased with age (13, 27.5, 34, and 41% at P35, P42, P49, and P60, respectively) (Supplementary Figure S5B).

To examine the potential functional consequences of DNA methylation changes during natural retinal degeneration, pathway analyses were performed on genes associated with DMRs. An average of 56% of pathways found were represented in all ages from P35-60, and 24% in P42-P60 (Supplementary Figure S6A). Pathway alterations were increasingly similar and more statistically significant with rat age between males and females (45%-P35, 61%-P42, 72%-P49, and 81%-P60) (Figure 3E; Supplementary Figure S6B). The top shared pathways identified via this approach were involved in neuronal and calcium signaling (Figure 3E), which were also found after TES treatment, indicating that TES affects similar pathways found in retinal degeneration. When comparing the pathways identified during retinal degeneration and TES treatment, an average of 45% of pathways overlapped between retinal degeneration and high-current TES samples from female rats as compared to 8% in males (Figure 3F). However, the degree of overlap was similar for both female and male rats in the low-current TES group (19 and 20% respectively). These results suggest that TES modulates some of the same pathways involved in retinal degeneration, with a more pronounced overlap in female rats exposed to high-current stimulation compared to males. This finding highlights potential sex-specific differences in how TES interacts with molecular pathways, particularly under higher stimulation conditions.

### Development of a DNA methylation score to measure retinal degeneration in TES-treated retinas

**Figure 4:**
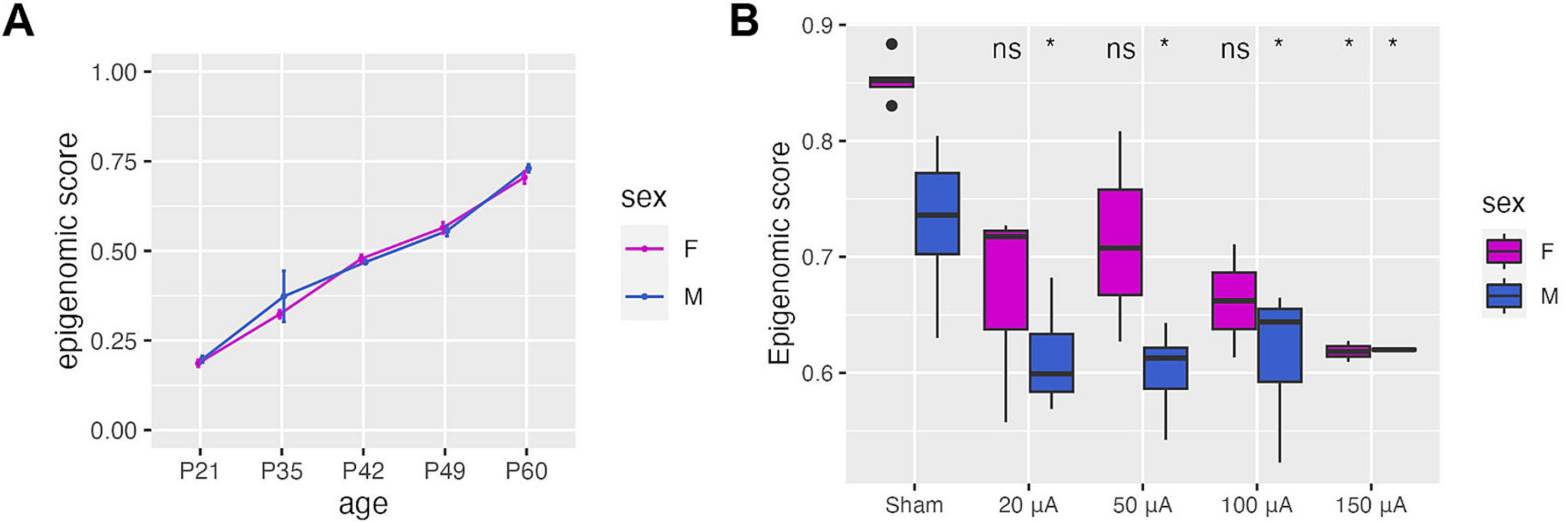
TES improves the epigenetic health of the retina. (A) Epigenomic scores derived from DMRs identified in the course of natural disease progression and TES treatment. This represents the epigenomic state of the retina. (B) Epigenomic scores calculated for sham and TES-treated retinas at P60, showing a reduction in epigenomic score values that coincide with retinal improvement. Asterisks denote a *p*-value <0.05 when compared to sham controls of the matching sex using Student’s *t*-tests.

To determine if TES-induced DNA methylation changes were associated with healthier, younger retinas in RCS rats, we developed a sex-specific epigenomic score based on a DNA methylation signature composed of the overlapping DMRs associated with both age-related retinal degeneration in untreated RCS rat retina and TES treatment (Supplementary Figure S6C). The epigenomic score increased linearly and equally with age in both male and female retinas in RCS untreated rats (Figure 4A), with a higher epigenomic score associated with a more degenerated retina. We then applied the epigenomic scores to TES-treated retinas and found that stimulated retinas had significantly lower epigenomic scores (*p*-value <0.05) compared to sham-treated or contralateral untreated retinas for both sexes (Figure 4B; Supplementary Figure S7). These findings support the ability of TES to improve retinal health in a manner that is reflected by epigenomic score changes.

### TES induces epigenomic changes in the superior colliculus that are similar to the retina

**Figure 5:**
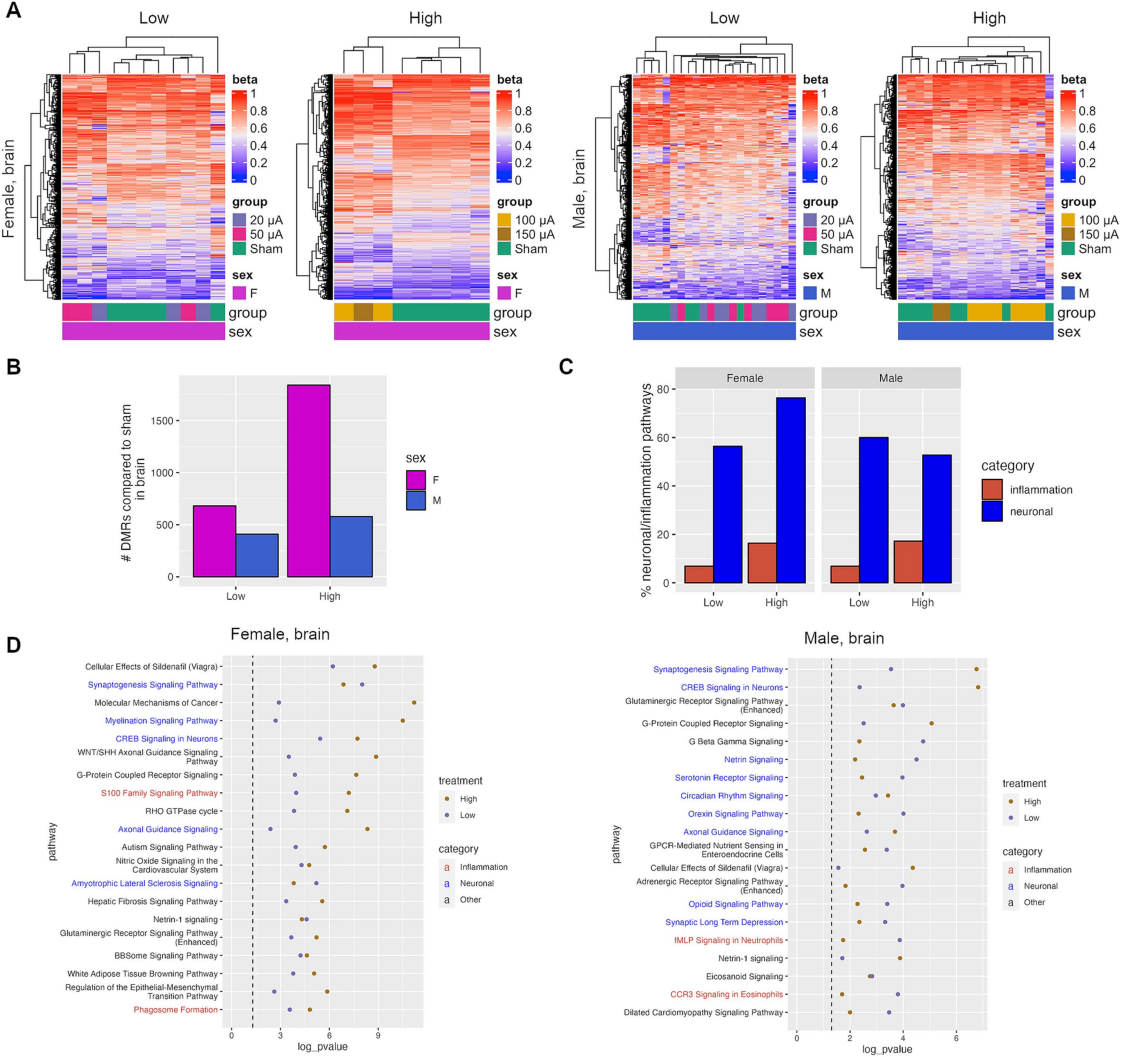
TES induces neuroprotective changes in the superior colliculus. (A) Heatmaps of DMRs identified in the superior colliculus of sham and TES-treated rats. (B) Number of DMRs identified in the superior colliculus. (C) Percent of all IPA pathways that fall under the neuronal or inflammation IPA pathway categories that were identified after TES treatment. (D) Top shared IPA pathways for all four treatment groups for females and males separately.

Studies have shown that retinal degeneration can lead to changes downstream of the retina in the visual processing regions of the brain (Bhattacharyya, 2022). To determine whether electrical stimulation affects the epigenomic profile of these brain regions, RRBS was performed on the superior colliculus, which is the brain region responsible for the integration of visual signaling and coordinating responses to visual cues. The superior colliculus was chosen over other brain regions, such as the lateral geniculate nucleus, due to its ability to retain residual visual input even in advanced stages of retinal degeneration. DNA methylation changes were identified between sham control animals and those in each treatment group (Figure 5A). TES-induced changes in the brain were sex-specific and positively correlated with current amplitude (Figure 5B), similar to what was observed in the retina (Figure 1D). Interestingly, a greater number of DMRs were identified in the brain compared to the retina, suggesting that TES may have a stronger epigenetic effect on the brain than the retina. This could be due to the benefits of TES being amplified as the visual signals travel from the retina to the brain. Additionally, in the superior colliculus, there were more unique DMRs found between each treatment group than were observed in the intersection between groups (Supplementary Figure S8A), or between the two sexes (Supplementary Figure S8B). This recapitulates what was seen in the retina.

Pathway analysis was performed on genes associated with DMRs identified in the superior colliculus. There was a high degree of overlap in pathways in the superior colliculus between treatment groups in both male and female rats (Supplementary Figure S8C). This is in contrast with the retina, where fewer overlapping pathways were identified in males. Similarly, more overlapping pathways were identified between females and males in the high current group, similar to the retina (Supplementary Figure S8D). Canonical pathways for genes within DMRs in the superior colliculus were investigated, which revealed an enrichment of neuronal protection and inflammation pathways similar to that observed in the retina (Figures 5C,D). Both low and high current amplitudes were associated with pathways linked to neuron function/maintenance compared with the sham group, including CREB signaling in neurons, axonal guidance signaling, or the synaptogenesis signaling pathway, which were enriched in both females and males (Figure 5D). These changes in many neuronal pathways were also treatment-dependent, suggesting that higher currents may induce more neuronal responses (Supplementary Figure S8E).

Overall, there was a strong concordance between the pathways identified in TES-treated retinas and those identified in the superior colliculus from the same animals, with an average of 75 and 48% of all significant pathways being shared in the high-current groups of female and male rats, respectively (Supplementary Figure S9A). This includes shared pathways such as the CREB signaling in neurons and axonal guidance pathways (Supplementary Figure S9B). This demonstrates that the impact of TES on the retina is mirrored in the superior colliculus of rats.

### TES-induced retinal DNA methylation changes are found in cfDNA

**Figure 6:**
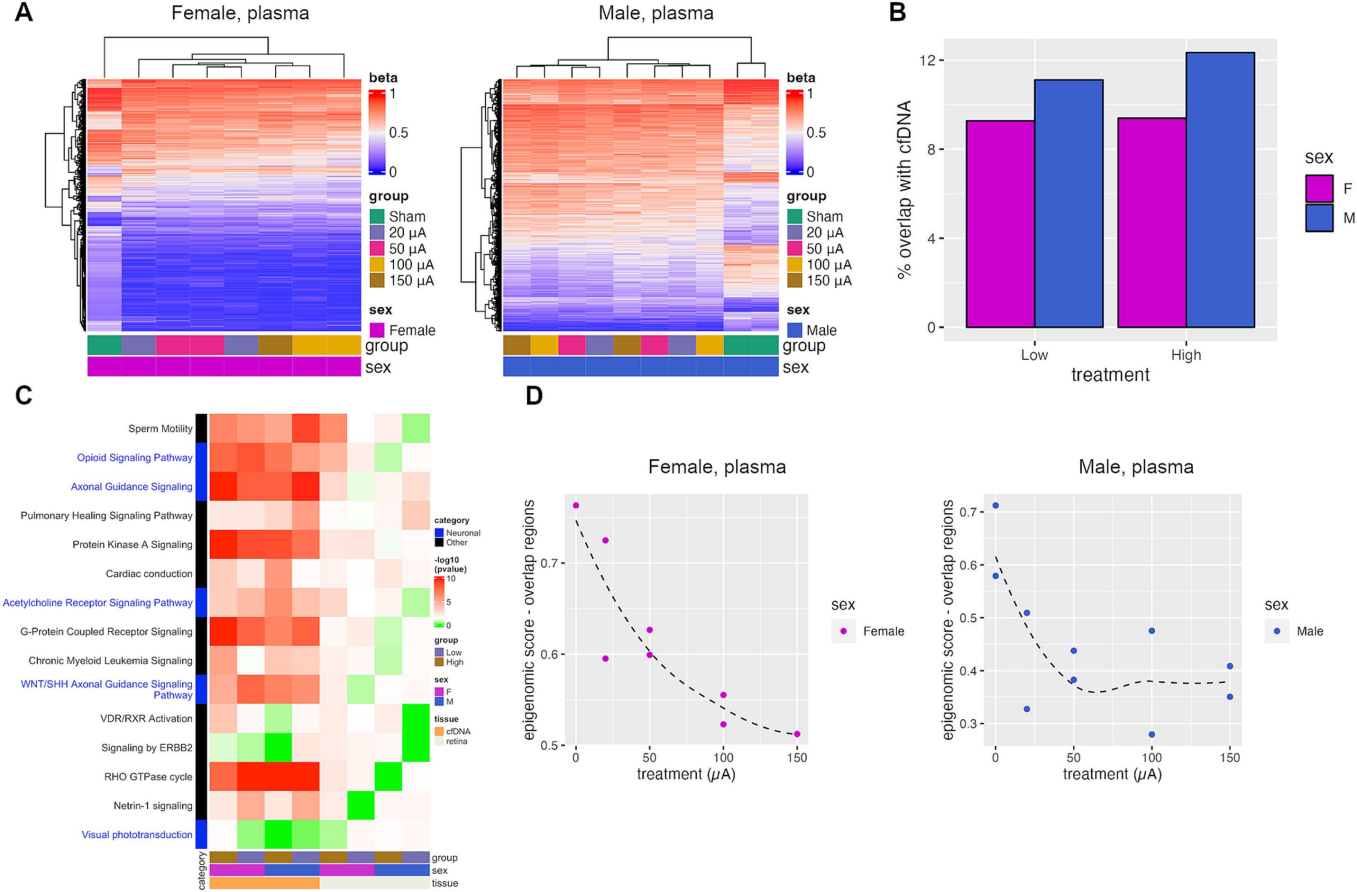
TES-induced DNA methylation changes are detectable in plasma cfDNA. (A) Heatmaps of DMRs identified when comparing plasma cfDNA collected from sham and TES-treated rats at P60. (B) Percent of DMRs identified in the retina tissue that were also identified in the plasma cfDNA. (C) *p*-values of top IPA pathways identified in retinal tissue that was also found in plasma cfDNA. Red indicates statistical significance (*p*-value <0.05), green indicates the absence of significance. (D) Epigenomic scores calculated from pools of cfDNA from each treatment group. The Loess regression curve is shown in the dotted line.

Cell-free DNA methylation is an emerging biomarker for monitoring disease changes and response to therapy. To determine if cfDNA methylation could potentially be used to monitor retinal degeneration and response to TES, we performed WGBS on plasma cfDNA from TES and sham-treated rats. As with retinal tissue, TES also induced cfDNA methylation changes (Figure 6A). In order to determine which methylation changes identified in the plasma cfDNA were also present in the retina, the overlap of the DMRs between the retina and the cfDNA was evaluated (Figure 6B). On average, 10.5% of the DMRs found in the retina were also found in the cfDNA. This degree of overlap is consistent with studies comparing methylation profiles of plasma cfDNA to tumor tissue (Legendre et al., 2015; Buckley et al., 2023). IPA analyses of the DMR-associated genes identified in cfDNA revealed that all of the top pathways previously identified in the retina were also found in the cfDNA for all treatment groups and for both sexes, including pathways such as axonal guidance signaling (Figure 6C). Increased statistical significance in the cfDNA compared to tissue was due to increased genomic coverage in WGBS compared to RRBS, which resulted in additional DMRs identified. However, the strong overlap between DMRs and pathways bolsters the case for the use of cfDNA methylation as a biomarker for monitoring disease status.

The epigenomic scoring algorithm for cfDNA samples from different treatment groups had to be modified, as not all methylation changes could be detected in the cfDNA. As such, only the subset of genomic regions that overlapped between the retina and plasma cfDNA were used to calculate the epigenomic score for the cfDNA (Figure 6D). There was an observable decrease in the epigenomic score with higher treatment intensities in females and in males (Figure 6D). In females, the epigenomic score decreased progressively with treatment intensity, whereas it plateaued at 50 μA in males. This shows that TES-induced DNA methylation changes that correlate with retinal degeneration can also be observed in cfDNA.

## Discussion

While the phenotypic effects of TES have been previously documented (Gonzalez Calle et al., 2023), the molecular mechanisms underlying these changes are not fully understood. In this study, we measured DNA methylation and gene expression in the retinas of male and female RCS rats in response to TES. We demonstrated that both sex- and amplitude-specific DNA methylation changes were associated with retinal health, as indicated by the reversal of epigenomic alterations linked to retinal degeneration, which we measured using an epigenomic score we developed. In other studies, TES has been shown to downregulate Bax and other pro-apoptotic genes (Tao et al., 2016; Willmann et al., 2011) and to induce the expression of neurogenic markers like Sox2 and Wnt in Müller cells (Yu et al., 2020). The DNA methylation changes observed in response to TES in this study were associated with neuronal signaling pathways, including those related to neuronal function and maintenance. This is consistent with the knowledge that the TES currents used in our study were designed to stimulate the retinal neurons directly (Kosta et al., 2021; Paknahad et al., 2021). In addition, many of the differentially methylated and expressed genes identified in this study were reported to be important in rod differentiation and development, such as *Nr2e3* and *Rho*. The reduced expression of *Nr2e3* observed in our study, for example, suggests a protective mechanism triggered in degenerating photoreceptor cells to ensure the survival of the retina under stress in RP (Rahman et al., 2020). Similarly, reduced expression of *Rho* might limit metabolic stress, allowing for the maintenance of retinal homeostasis to prevent the degeneration of photoreceptor cells (Moore et al., 2020; Athanasiou et al., 2018). As a result, inhibiting the *Nr2e3 and Rho* pathways has been suggested as a potential neuroprotective strategy to treat RP (Moore et al., 2020), and may also underlie a therapeutic mechanism through which TES exerts its benefits.

As bulk DNA methylation and RNA sequencing was performed on the retinas, some of the observed changes could be influenced by the alterations in cell proportions that occur during retinal degeneration, such as reduction in photoreceptors, or TES treatment. Since the entire retina was used for sequencing, we were unable to determine the proportion of each retinal cell type in the sample, or normalize DNA methylation and gene expression results based on the proportions. However, we have previously shown TES treatment at 100 μA increases photoreceptor survival by 38.3% (Gonzalez Calle et al., 2023), while our current studies show gene expression changes exceeding two-fold differences. This suggests that the molecular changes we observed, such as downregulation of *Nr2e3* and *Rho*, were not likely solely due to a reduction in rod cells. Further validation would be required to definitively distinguish if the changes in gene expression were due to proportion changes, or changes within the retinal cell types, such as by immunohistochemistry or single-cell RNA sequencing.

TES has been shown to exert effects on the brain, even when the electrical fields have been confined to the eye (Agadagba et al., 2023; Gonzalez Calle et al., 2023). This is due to the preservation of retinal ganglion cells and preservation of healthy neuronal connections to the brain (Henrich-Noack et al., 2013). Our results identified DNA methylation changes in the superior colliculus of TES-treated rats, suggesting that there was an indirect effect of TES on the brain, the mechanisms where are unknown. Surprisingly, we also found a modest effect of TES on the unstimulated contralateral eye. Computation modeling that we have previously performed shows that TES delivered by our setup is confined to one eye, suggesting that there would be no direct effect on the contralateral eye (Gonzalez Calle et al., 2023). This suggests that the TES may exert indirect molecular effects on the retina of the contralateral eye. Indeed, this phenomenon of TES affecting the contralateral eye has been observed in other studies (Wagner et al., 2023). Further studies will be required to better understand the underlying mechanisms of the observed effects of TES on the contralateral eye.

The relationship between DNA methylation and gene expression changes is complex and often dependent on the site of methylation (Jones, 2012), therefore, drawing conclusions from DNA methylation changes is not always straightforward. Epigenetic alterations, specifically in DNA methylation, has been shown to play an important role in natural retinal development and in retinal degeneration (Lu et al., 2020) and can be progressive in nature (Bocklandt et al., 2011). To better understand the relationship between DNA methylation changes and retinal health in our study, we developed an epigenomic score for retinal degeneration based on the observed progressive methylation changes. This score, which increases with degeneration, is not dependent on understanding the exact function of individual methylation changes but instead tracks a set of changes that parallel retinal health.

While the DMRs observed in both the retina and the brain had minimal overlap between TES current amplitudes and sexes, the pathways affected by these changes were consistent. This suggests that TES may not have a targeted mechanism of action by inducing methylation at specific loci, but rather induces global methylation changes that converge on specific signaling pathways. This is supported by studies showing the semi-stochastic effect of electrical fields on neuronal activity (Formento et al., 2020), and the ability of electrical fields to induce methylation changes on a global level (Liu et al., 2015; Ashok et al., 2023). Electrical fields have been shown to modulate DNA methyltransferases (DNMTs), which regulate global DNA methylation levels (Liu et al., 2015), and this may account for the TES-induced epigenetic changes observed in this study.

Females have been found to be more susceptible to ocular diseases as compared to males (Zetterberg, 2016; Aninye et al., 2021; Korpole et al., 2022). These sex-related differences have also been characterized in animal models of retinal degeneration. The degeneration of cone cells is more rapid in female rd10 mice (Li et al., 2019). Several causes of these sex-specific effects have been proposed, with differences in hormonal regulation being a particularly prominent factor (Korpole et al., 2022). In our experimental system, untreated female RCS rats had higher overall epigenomic scores compared to males, suggesting that they had more degenerated retinas at P60. However, females also presented with a stronger response to TES in terms of observed DNA methylation and pathway changes, which also correlated with lower epigenomic scores after treatment compared to males. A potential explanation for the stronger responses observed in females may be related to their higher levels of estrogen, which is known to have neuroprotective effects (Kaja et al., 2003). The neuroprotective effects of estrogen could potentially augment the effect of TES.

Blood biomarkers are commonly employed in multiple diseases as a non-invasive means of monitoring progression and treatment efficacy. Specifically, the analysis of plasma cfDNA methylation has been shown to be a feasible approach to the detection of cancer (Buckley et al., 2023; Guo et al., 2021). For retinal degenerative diseases, proteomic and metabolomic changes in blood and ocular fluids have reported and show potential as biomarkers of disease progression (Oca et al., 2021; Lains et al., 2018). Differential methylation of plasma cfDNA has been identified in AMD patients, but the markers found were not sufficiently strong to be further developed (Oliver et al., 2015). Our epigenomic score, comprising a DNA methylation signature derived from degenerated rat retinal tissue, can also be utilized in plasma cfDNA. While this signature was developed from the RCS rat model, it is worthwhile to determine if the same plasma cfDNA biomarkers can be applied to other rodent models of retinal degeneration. This approach also requires validation and testing in humans, as translating the findings is complicated by differences between the rat and human methylomes. Additionally, not all animals in this study and our previous study (Gonzalez Calle et al., 2023) responded equally to TES treatment, suggesting that this methylation signature could potentially be used to predict retinal response to TES therapy.

In summary, we have demonstrated in a well-established rodent model of retinal degeneration that non-invasive TES induces sex- and current amplitude-specific changes in patterns of DNA methylation. These changes were correlated with gene expression changes associated with induction of neuroprotective effects. The epigenomic score developed in this study was used as a molecular indicator of retinal health, which was also assessed using cfDNA samples. These novel findings are highly translational and hold potential to enhance the management of degenerative eye diseases by guiding the selection and clinical implementation of key biomarkers.

## Data Availability

Data generated in this work has been deposited in NCBI Sequence Read Archive (SRA) under BioProject ID PRJNA1190390.
