# Supplementary material for: Transcorneal electrical stimulation restores DNA methylation changes in retinal degeneration
Source: Front Mol Neurosci. 2024 Dec 5;17:1484964. doi: 10.3389/fnmol.2024.1484964 (PMC11656077; doi:10.3389/fnmol.2024.1484964)
Supplement: Supplementary file 1 [file Data_Sheet_1.PDF]

Supplementary Materials

Supplementary Figures

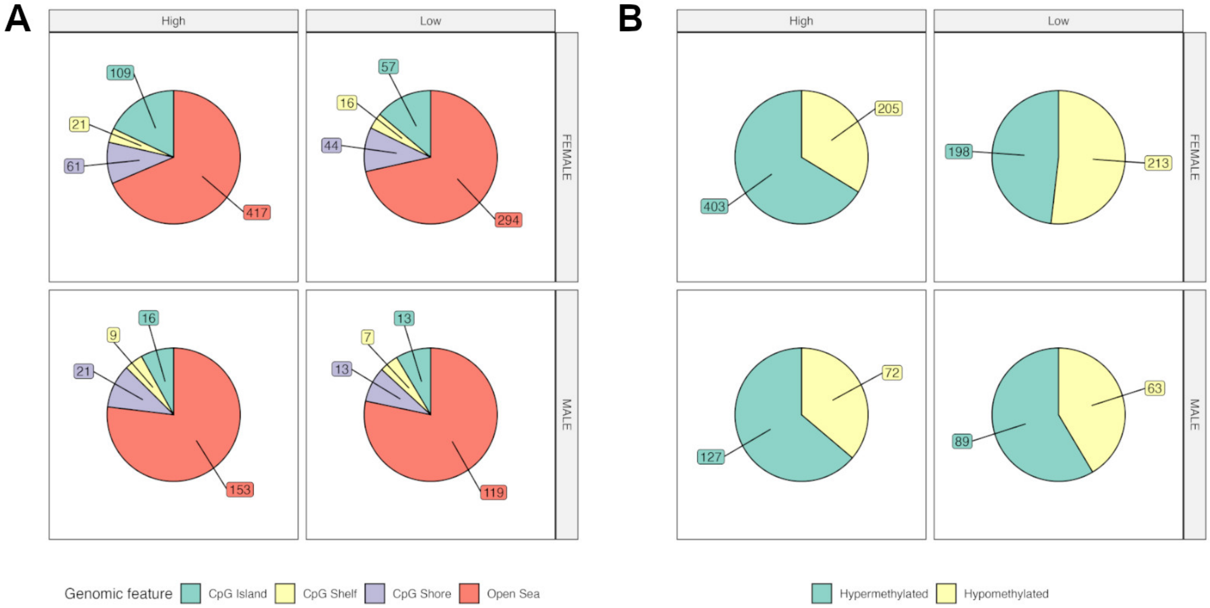

Figure S1. Properties of TES-induced differentially methylated regions. Pie charts showing (A) the distribution of hyper- and hypomethylated DMRs, and (B) the distribution of DMRs on CpG islands, shores and non-CpG regions.

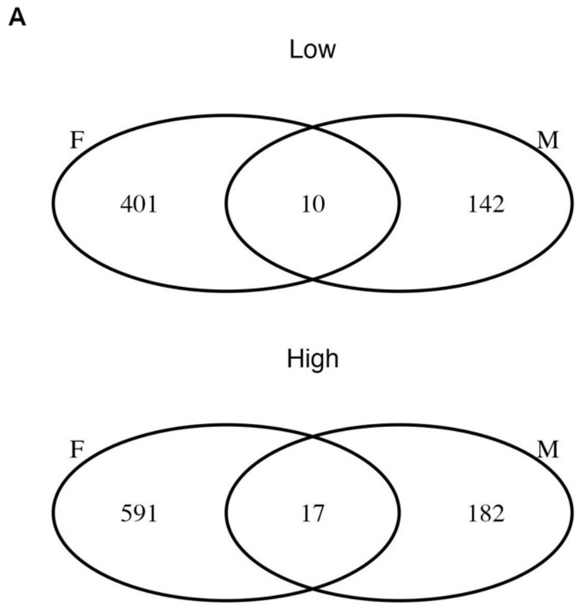

Figure S2. Overlapping DMRs identified in the retina after TES-treatment. (A) Venn diagram showing the overlap between DMRs in the retina for females and males, for the high and low treatment groups separately, and (B) overlap between high and low treatment groups, for females and males separately.

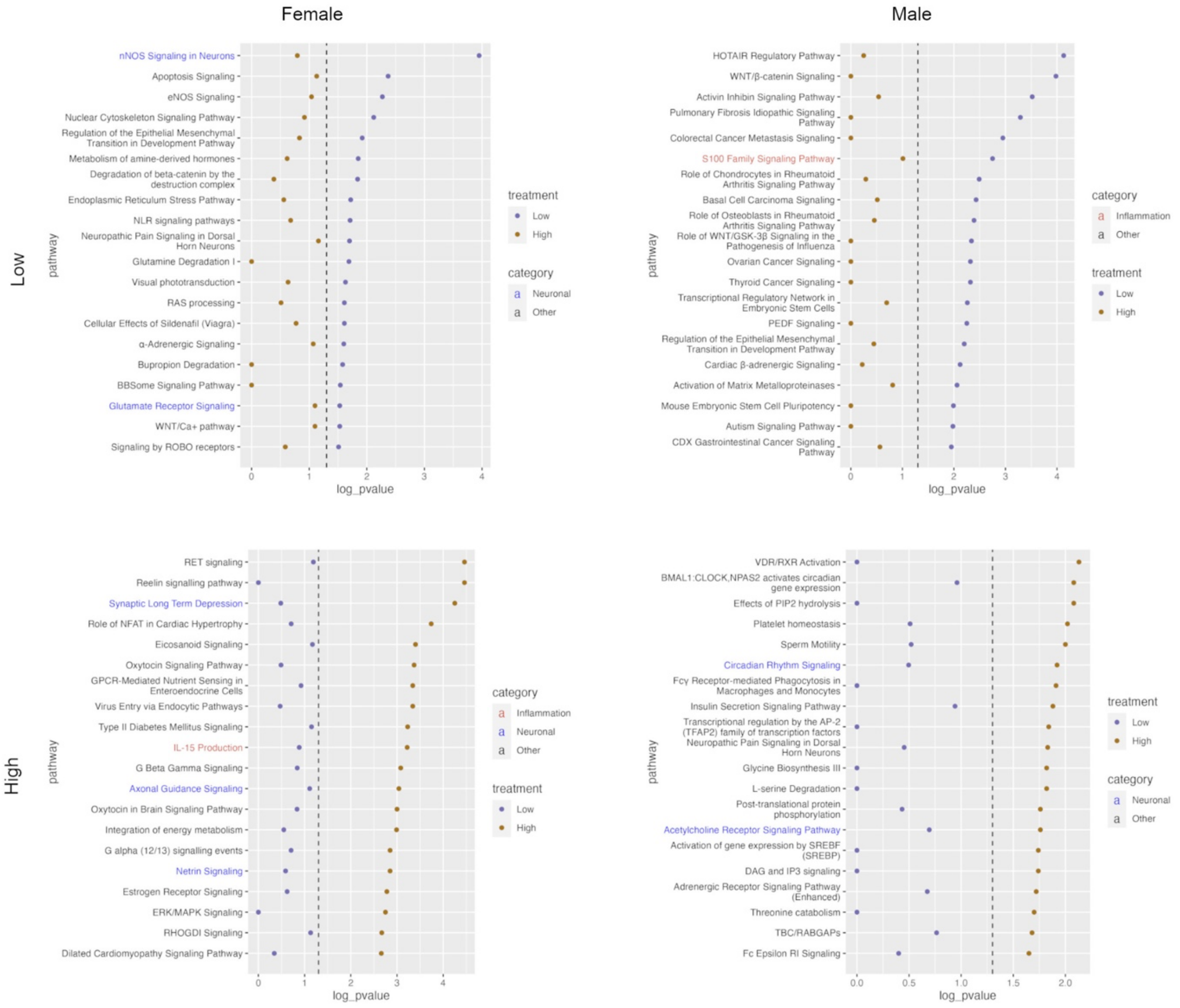

Figure S3. Top IPA pathways unique to each treatment group. Top 20 IPA pathways that were unique in low (top panel) or high (lower) treatment groups for females (left column) and males (right column).

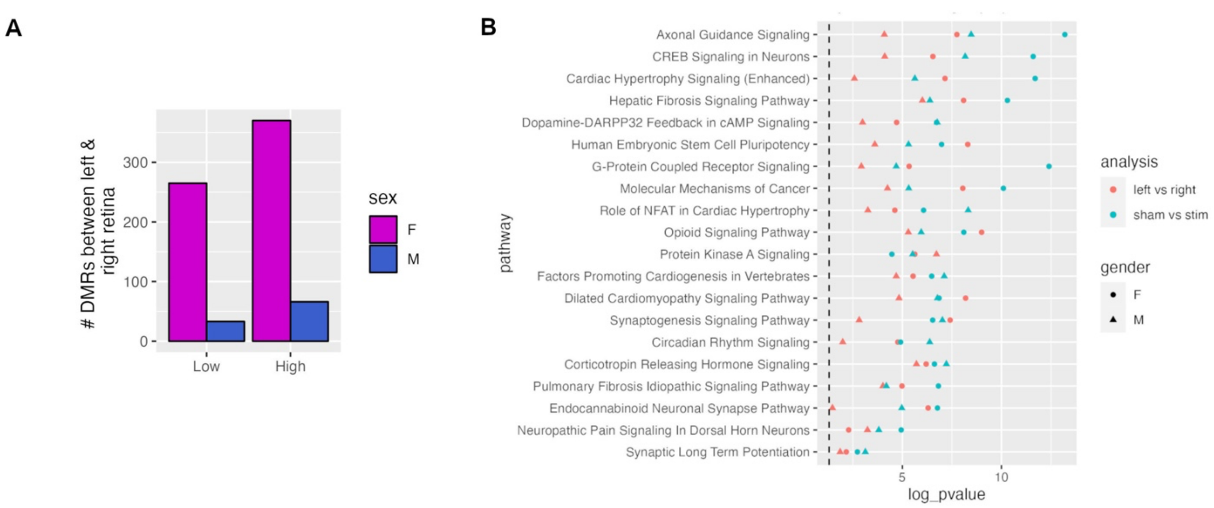

Figure S4. TES induces similar DNA methylation changes when compared treated to untreated retina. (A) The number of DMRs identified between the untreated left retina and treated right retina of each rat. (B) Pathway analysis was performed on genes associated with DMRs for all current amplitudes (20-150 μA) and the mean p-value across all amplitudes was calculated. Top significant pathway changes identified between left and right retinas were similar to those identified between sham and stimulated retinas.

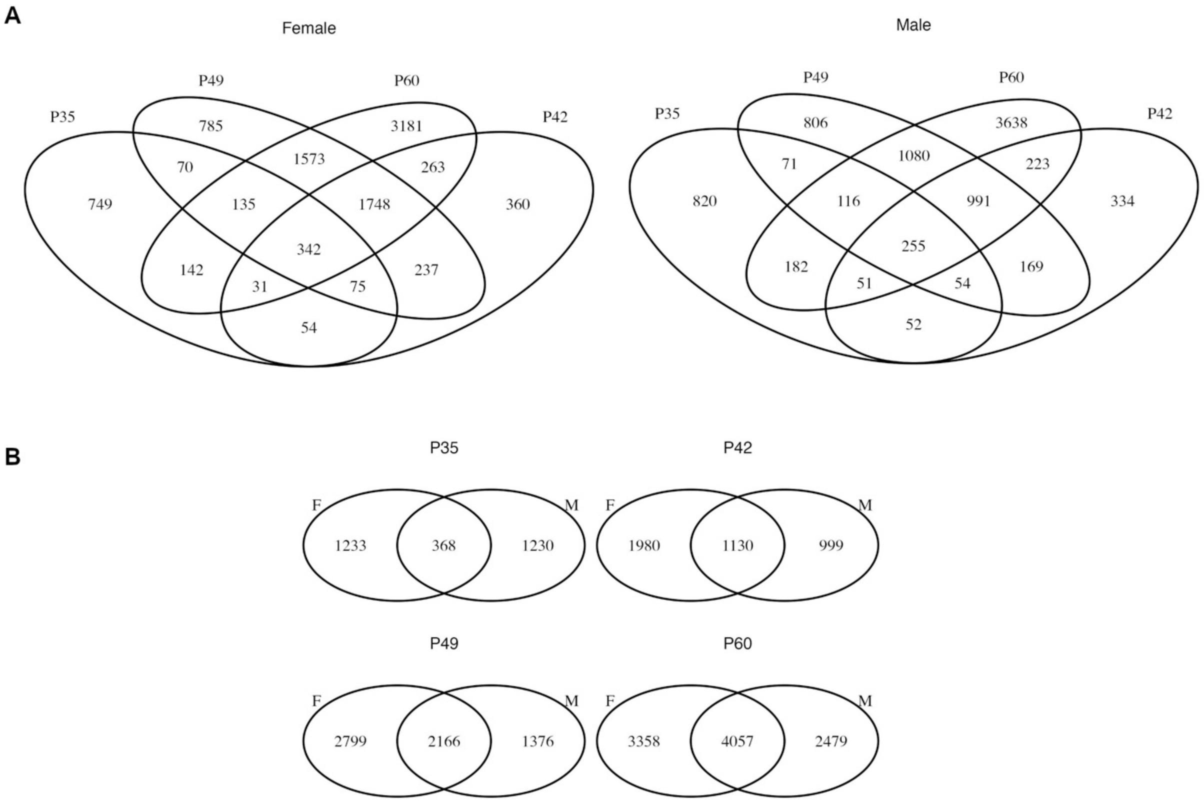

Figure S5. Overlapping DMRs during natural retinal degeneration in RCS rats. (A) Venn diagram showing the overlap between DMRs when comparing P21 rats to each age group. (B) Venn diagram showing the overlap between DMRs identified in females and males for each age group.

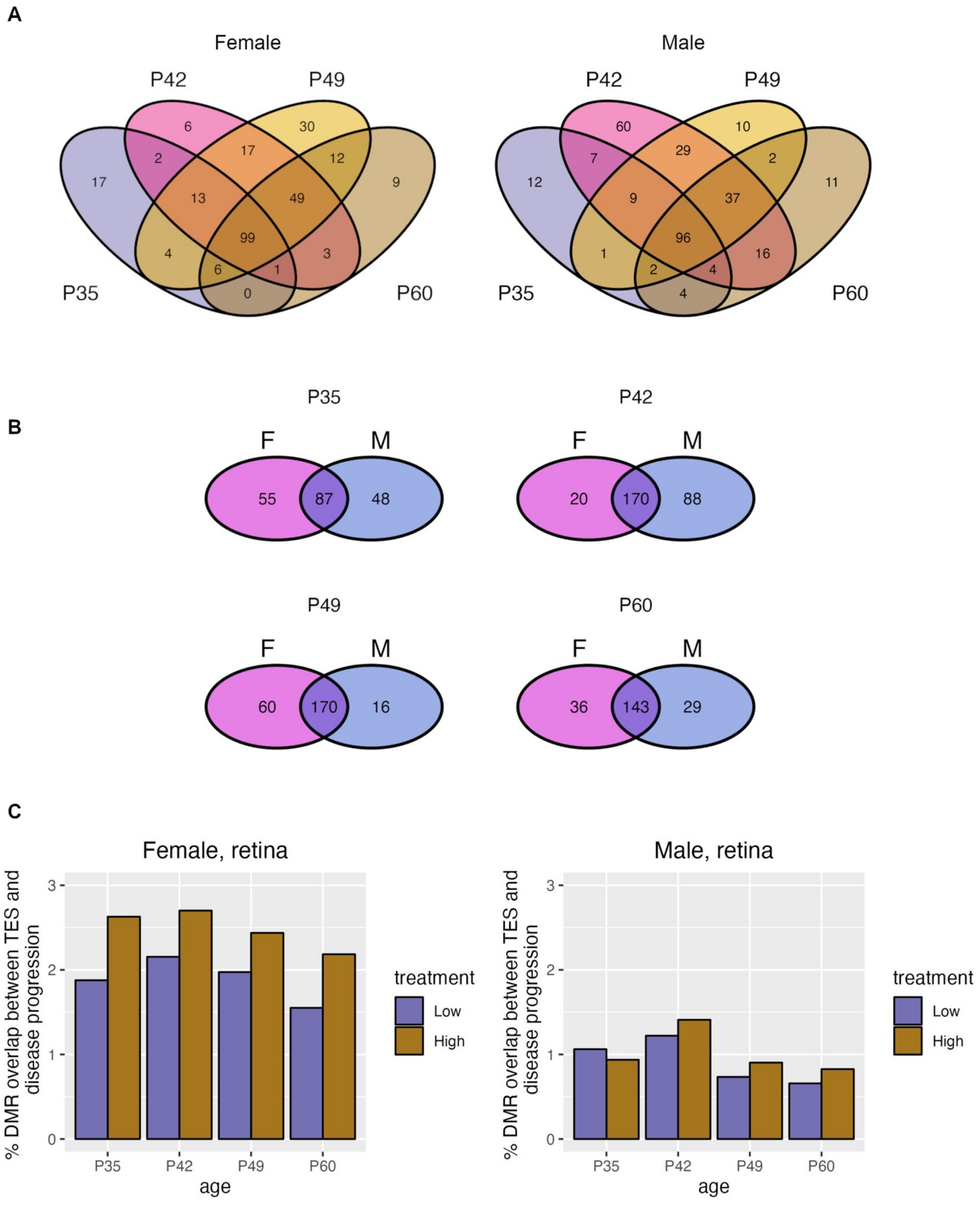

Figure S6. Shared and unique IPA pathways during natural retinal degeneration. (A-B) Venn diagram showing IPA pathways that were common and unique between (A) each age group for females and males separately, or (B) females and males for each age group. (C) Percent of DMRs identified during natural retina degeneration that were also found after TES treatment.

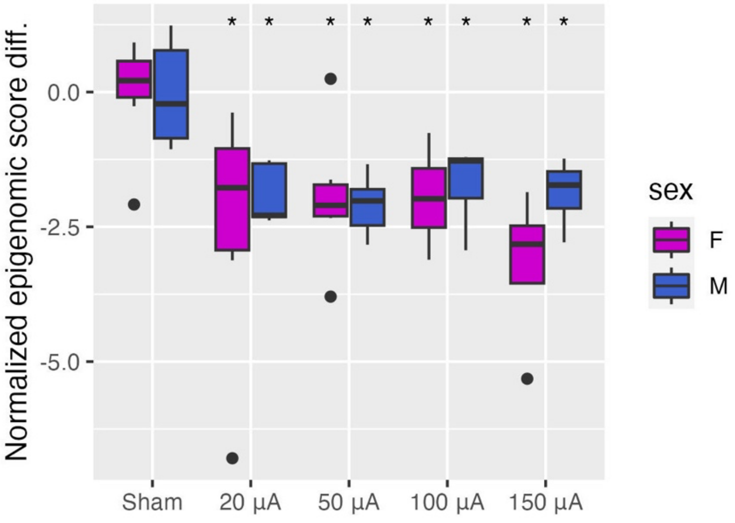

Figure S7. TES improved epigenetic health of retinas when comparing untreated to treated eye. Difference of epigenomic scores of between the left and right retinas. The scores were normalized using a z-score approach where the mean score was 0 for the sham groups.

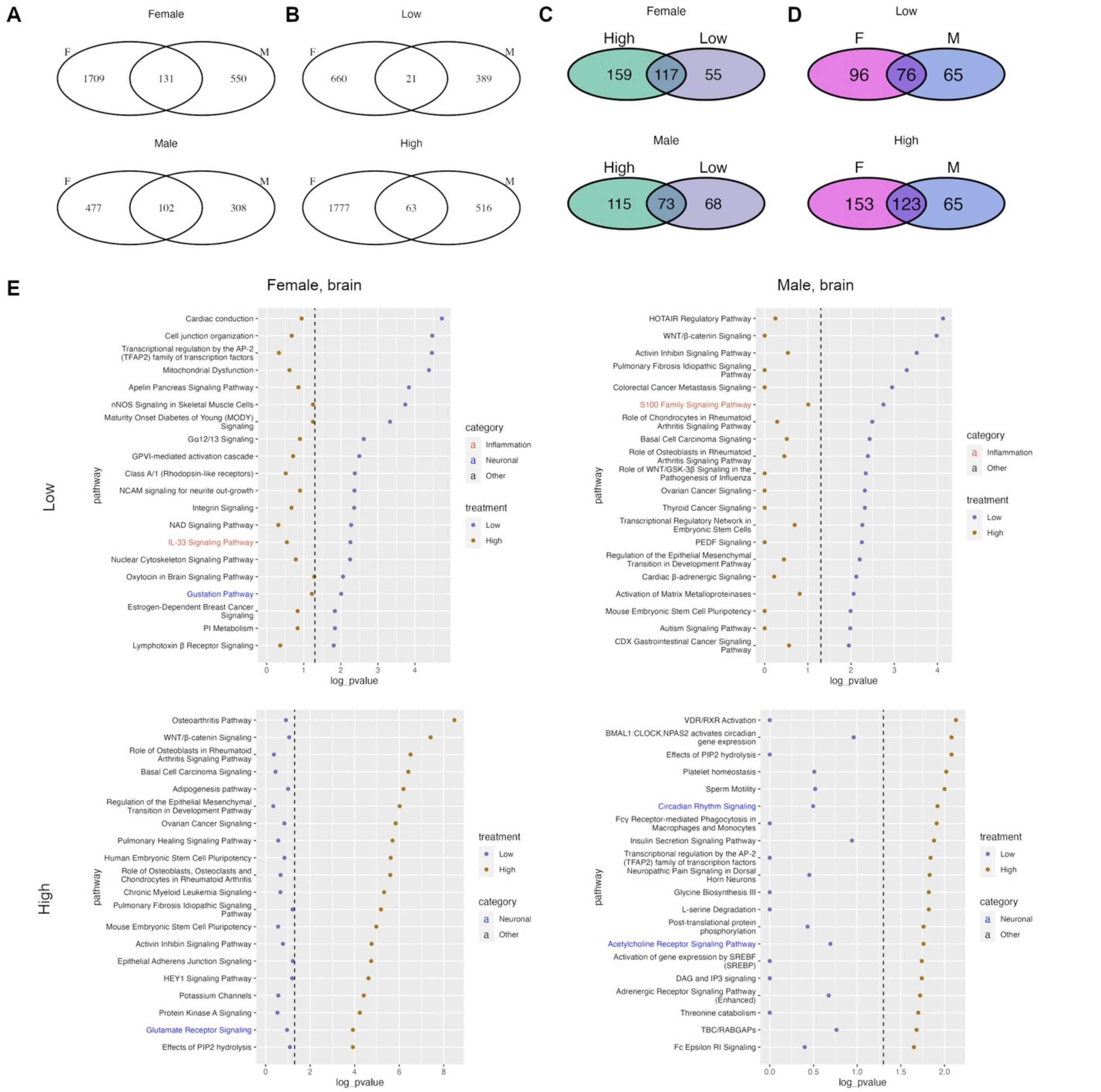

Figure S8. DMRs and pathways identified in the superior colliculus after TES-treatment. (A) Venn diagram showing the overlap between DMRs in the superior colliculus when comparing sham to the different treatment groups for females and males. (B) Venn diagram showing the overlap between DMRs identified between females and males for each treatment group. (C) Venn diagram of common and shared IPA pathways identified from DMRs in the superior colliculus for each treatment group. (D) Venn diagram of IPA pathways that were common or unique to females or males for each treatment group in the superior colliculus. (E) Top 20 IPA pathways that were significant in either high or low treatment groups, for females and males separately.

A

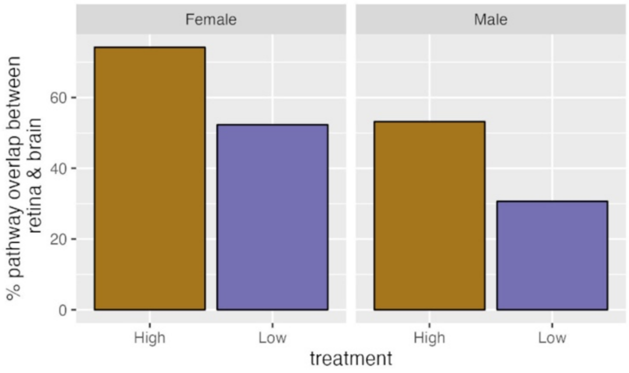

B

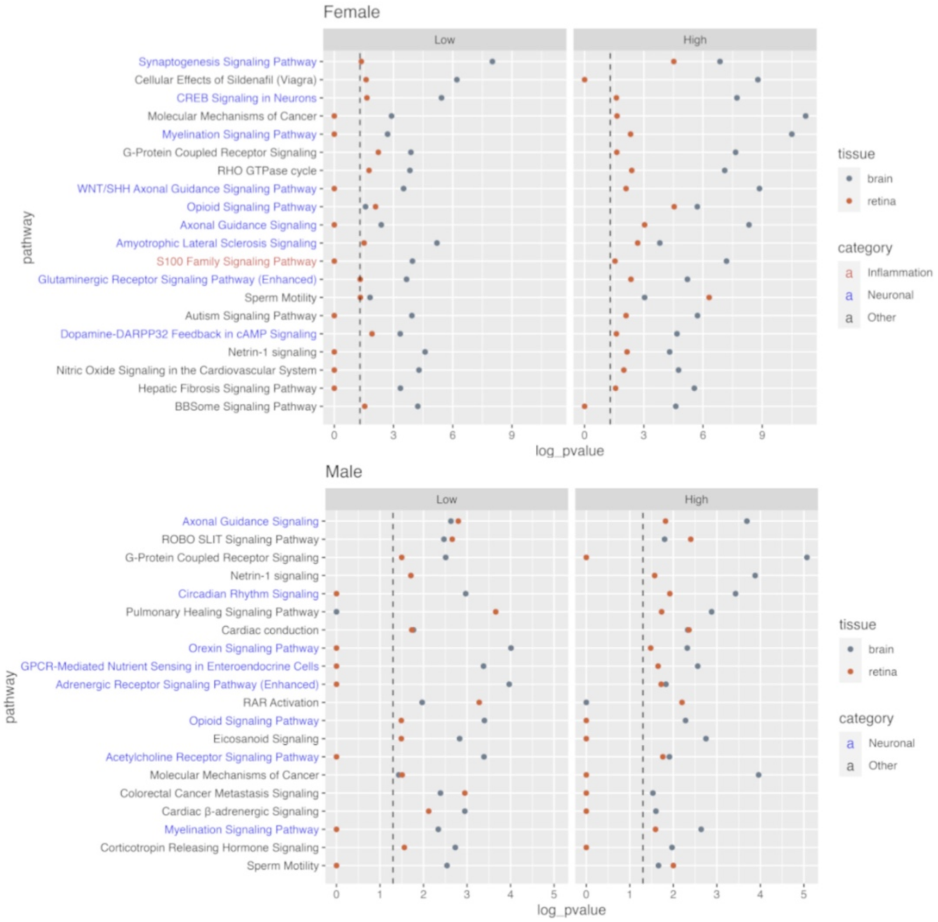

Figure S9. IPA pathways shared between retina and superior colliculus (A) Percent of significant pathways identified in the retina that were also found in the superior colliculus**.** (B) Top IPA pathways that were found in both retina and superior colliculus in most treatment groups, for females and males separately.

Supplementary Tables

Treatment	Males	Females
Sham	7	5
Low (20-50 μA)	14	6
High (100-150 μA)	10	4

Table S1. Number of TES and sham-treated RCS rats

Age	Males	Females
P21	8	4
P35	4	3
P42	4	4
P49	4	4
P60	4	4

Table S2. Number of untreated RCS rats collected at each time point

Treatment	Males	Females
Sham	2 pools (n=3,4)	1 pool (n=3)
20 μA	2 pools (n=3,3)	2 pools (n=3,4)
50 μA	2 pools (n=3,4)	2 pools (n=3,3)
100 μA	2 pools (n=2,4)	2 pools (n=3,3)
150 μA	2 pools (n=3,3)	1 pool (n=3)

Table S3. Number of cfDNA pools, and the number of RCS rats per pool.
